# Evaluating osseointegration, bone remodeling and osteolysis after total wrist arthroplasty using photon-counting detector CT

**DOI:** 10.1007/s00256-026-05276-2

**Published:** 2026-06-03

**Authors:** Nina Kämmerling, Simon Farnebo, Gustav Lind, Mårten Sandstedt, Anders Persson, Erik Tesselaar

**Affiliations:** 1 Clinical Department of Radiology in Linköping, Region Östergötland, Linköping, Sweden; 2 Center for Medical Image Science and Visualization (CMIV), Linköping University, Linköping, Sweden; 3 Department of Health, Medicine, and Caring Sciences, Linköping University, Linköping, Sweden; 4 Clinical Department of Medical Radiation Physics, Region Östergötland, Linköping, Sweden; 5 Clinical Department of Hand Surgery, Plastic Surgery and Burns in Linköping, Region Östergötland, Linköping, Sweden; 6 Department of Biomedical and Clinical Sciences, Linköping University, Linköping, Sweden

**Keywords:** Implant, Prosthesis, Computed tomography, Wrist joint, Bone quality, Osteolysis, Cortical thickness, Cortical density, Osseointegration

## Abstract

**Objective:**

To evaluate osseointegration, bone quality, and periprosthetic osteolysis following total wrist arthroplasty using photon-counting detector CT (PCD-CT).

**Materials and methods:**

Nineteen patients with total wrist arthroplasty were examined with PCD-CT the day after surgery, at 6 and 12 months postoperatively. CT images were reviewed and osseointegration and periprosthetic osteolysis were evaluated. Cortical thickness and cortical density were measured. The bones around the implant were divided into zones, and the evaluations were made in each zone.

**Results:**

Osseointegration, visualized as bone ingrowth to the implant, increased in all zones except for the capitate during the 12-month follow-up period. The highest percentage of osseointegration was found in the zones most distant from the joint space, with more than 50% of the circumference of the implant in 84.2% of the patients at the metacarpal screw tip, and in 89.5% of the patients at the radial screw tip at 12 months. Cortical thickness and cortical density decreased in radial distal zones. Osteolytic changes were observed in all but one patient, and increased during the follow-up period, starting near the joint space.

**Conclusion:**

Progressive osteolytic changes, and reduction in cortical thickness and density were observed during 12 months after total wrist arthroplasty, especially near the joint space. Concomitantly, continued osseointegration of the implants was observed on PCD-CT images. Osseointegration counteracts the diminished bone contact following osteolysis and may be an important factor to preserved stability.

## Introduction

Total wrist arthroplasty (TWA) is associated with relatively high complication rates, and implant survival at 10 years has repeatedly been reported around 80%, which is lower than for total hip and knee replacement. Newer fourth-generation bone-sparing techniques show more promising results; however, periprosthetic osteolysis and implant loosening remain the most common complications [1–4].

The cause of osteolysis is probably multifactorial, driven by inflammation, imbalance in bone remodeling, and mechanical stress and micromotion from the implant [5–7]. Inflammatory cascades can be triggered by tissue damage and wear particles [5, 8–10]. An altered distribution of biomechanical loading following prosthesis surgery can cause bone atrophy in areas with reduced strain, a phenomenon called stress shielding [11, 12]. Micromovements may increase fluid pressure at the interface between bone and implant and contribute to periprosthetic osteolysis [6, 13]. Whatever the origin might be, failed osteointegration and osteolysis are two of the main causes of implant failure even though the extent of osteolytic changes does not correlate very well with subsequent implant failure and patient symptoms, and the osteolytic process may even stabilize years postoperatively [14–16].

Follow-ups of TWA patients often include conventional radiographic images. However, the method has limitations in overprojection of structures, which may obscure subtle bone changes, and does not allow for a detailed visualization of bone microarchitecture. Conventional energy-integrating CT has not been an optimal alternative due to metal artifacts making it difficult to assess the bone–metal interface, most important in diagnosing osteolysis and implant loosening. In recent years, PCD-CT has become available at many larger clinical radiology departments. The relatively new CT technique enables a higher spatial resolution at lower radiation doses, improved metal artifact reduction in postoperative patients, and simplified assessment of bone quality [17]. This makes the technique potentially suitable for improved visualization of bone microstructure, which is important for detection and assessment of osteolytic changes, but also for assessment of osseointegration and implant stability [18–22].

The purpose of this study was to evaluate bone quality changes and periprosthetic osteolysis. We also aimed to assess signs of osseointegration which, to our knowledge, have not yet been described in patients following TWA using PCD-CT.

## Materials and methods

### Patients

Nineteen patients with total wrist arthroplasty were included in the study, which was approved by the Swedish Ethical Review Authority (Dnr. 2021-02034). Inclusion criteria were as follows: total wrist arthroplasty using Motec® Wrist Joint Prosthesis (Motec, Swemac Innovation AB, Linköping, Sweden), adult, non-pregnant, ability to participate, and provision of oral and written informed consent.

**Fig. 1:**
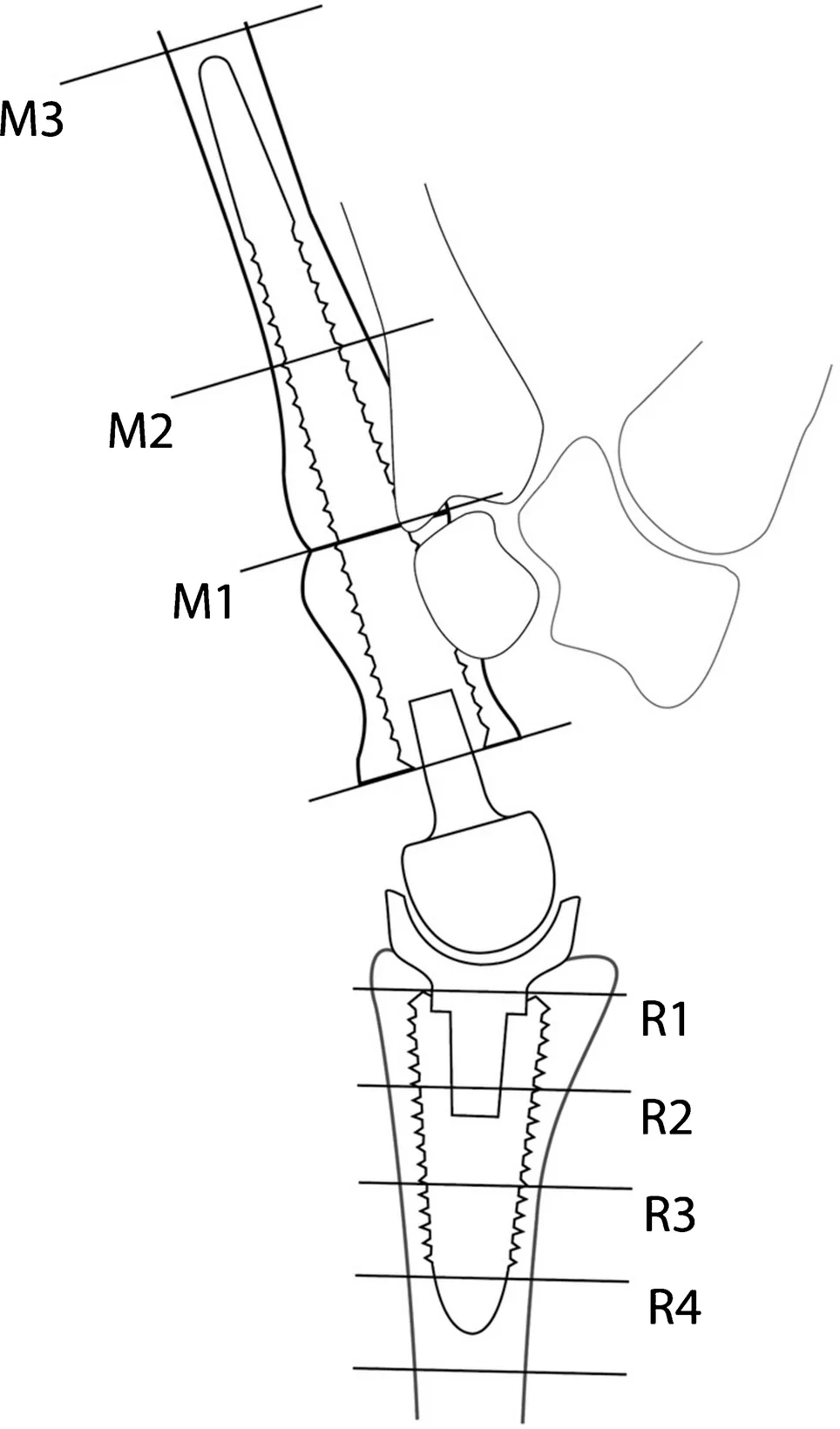
Schematic illustration of implant and zones. The bone around the radial screw was divided into four zones, which were named as follows: R1 – the most distal radial zone; R2 – the second most distal radial zone; R3 – the second most proximal radial zone; R4 – the most proximal radial zone comprising the stem tip. The bone around the distal screw was divided into three zones: zone M1 – capitate; zone M2 – the base of the third metacarpal; zone M3 – diaphysis of the third metacarpal

PCD-CT examinations were performed the day after surgery, and at 6 and 12 months postoperatively. The bones into which the implants were fixated, were divided into zones adapted from Chakrabarti et al. [23] for subsequent evaluation (Fig. 1).

#### Surgery

The Motec Wrist Joint Prosthesis is an uncemented modular arthroplasty system that uses a ball-and-socket articulation. It is fixated in the radius proximally, and the capitate and middle metacarpal distally through conical-shaped titanium alloy screws. The first carpal row (scaphoid, lunate and triquetrum) is removed during surgery to create space for the implant. Screw fixation is achieved through a tight fit of the screws to the cortical bone of the distal radius and at the isthmus level of the third metacarpal. The ball component is secured in the distal screw, and the cup component in the proximal screw. The cup is lined with ultrahigh-molecular-weight-polyethylene (UHMPE). After 2 weeks of plaster postoperatively, the patients were allowed to move their hands using a structured rehabilitation program. Full motion and weight-bearing was allowed 8 weeks after surgery.

##### Imaging protocol

All examinations were performed on a photon-counting detector CT system (NAEOTOM Alpha, Siemens Healthineers, Forchheim, Germany) using the same clinical ultra–high-resolution protocol. Scans were acquired at 140 kVp with tin prefiltration, collimation 120 × 0.2 mm, pitch 0.8, and rotation time 1.0 s. The volume CT dose index (CTDI_vol_) was 7.3 ± 0.3 mGy, which is similar to the radiation dose used in our clinical protocol for the EID-CT system.

Low-threshold polyenergetic (T3D) images with a sharp kernel (Br89), as well as virtual monoenergetic images (VMI) at a level of 130 keV (kernel Br76) were reconstructed with quantum iterative reconstruction (QIR) strength 3. Reconstructions were generated on a 1024 × 1024 matrix with a patient-dependent field of view (62–183 mm). Axial images were reconstructed at 0.2 mm (increment 0.1 mm) and 1.0 mm (increment 0.5 mm); axial VMI 130 keV images at 0.4 mm (increment 0.2 mm). Coronal and sagittal reformations were reconstructed at 1.0 mm. Additional multiplanar reformations were created as needed at reader-preferred slice thicknesses (Table 1). The protocol was based on our prior optimization work and clinical experience with postoperative wrist imaging using PCD-CT.

**Table 1:** Acquisition and reconstruction parameters

Tube voltage/prefiltration (kVp)	Sn140
Scan mode	Quantum HD Sn
Collimation (mm)	120 × 0.2
Pitch	0.8
Rotation time (s)	1.0
CTDI_vol,32_ (mGy, mean ± SD)	7.3 ± 0.3
Image reconstructions	Low threshold (T3D) VMI 130 keV
Reconstruction kernel	Br89 for T3D; Br76 for VMI
Iterative reconstruction strength	QIR3
Slice thickness/increment (mm)	0.2/0.1; 1.0/0.5 (T3D)
	0.4/0.2 (VMI 130 keV)
Field-of-view (mm)	62–183 (patient-dependent)
Reconstruction matrix	1024 × 1024

#### Assessments of osseointegration and osteolysis

Osseointegration was defined as direct contact between the surface of the implant and cortical bone, or as bone ingrowth with bone tissue formed in the medullary cavity visibly connecting the surface of the implant to the cortical bone. The degree of osseointegration in each zone was estimated: 0%, > 0–25%, > 25–50%, or > 50%. PCD-CT images were reviewed independently by two readers, one consultant radiologist with more than 20 years of radiology experience, and one radiologist with 9 years of radiology experience.

**Fig. 2:**
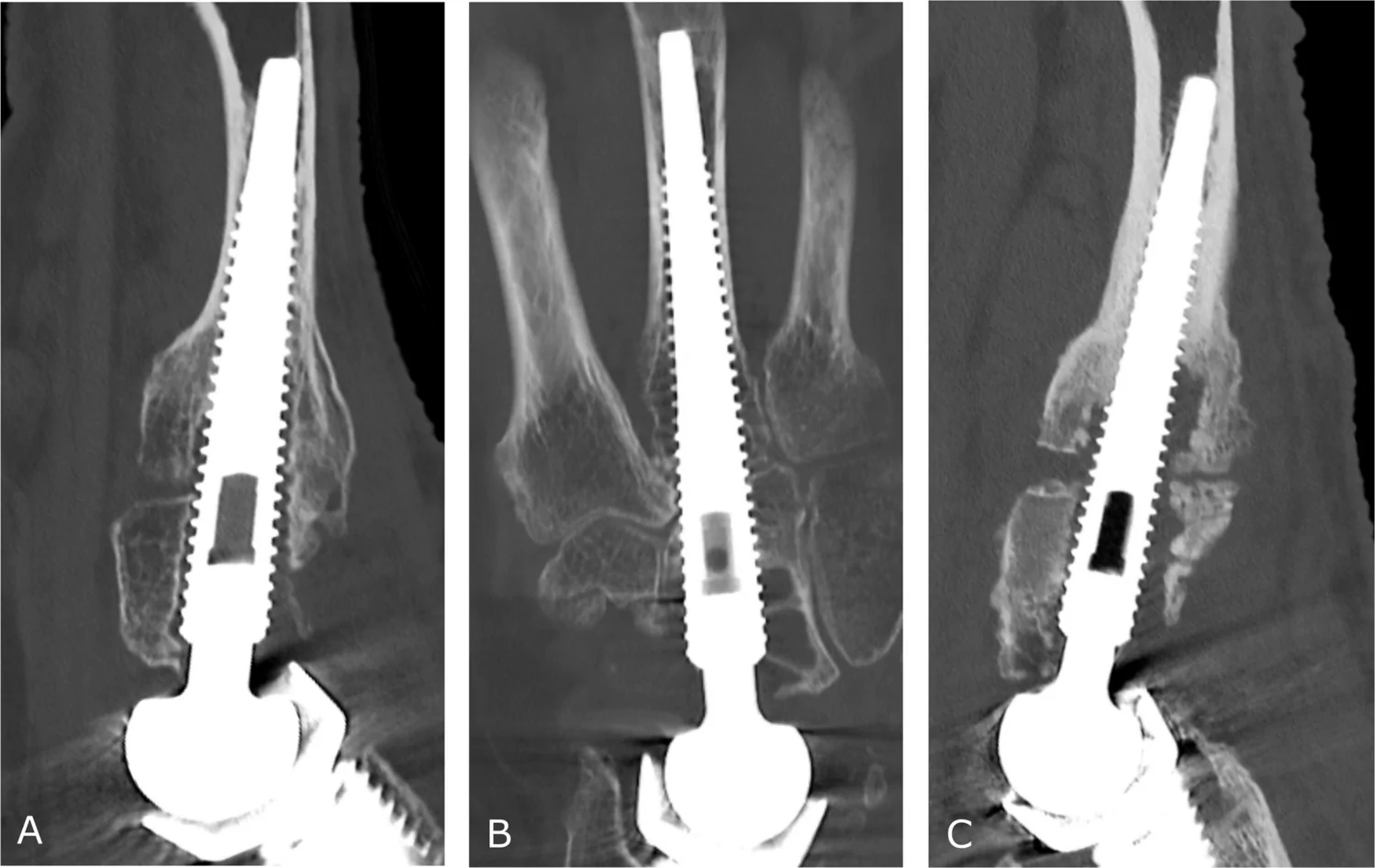
Different kinds of osteolytic changes. **A** Linear osteolysis around the screw in the capitate and in the base of the third metacarpal. **B** Cystic osteolysis in the capitate. **C** Mixed osteolysis in the capitate and in the base of the third metacarpal

Osteolytic changes were divided into linear, cystic (sometimes called expansile or scalloping/scalloped), or mixed. Linear osteolysis was defined as the presence of relatively uniform radiolucent zones at the interface between bone and implant, and cystic when there was a rounded, sharply demarcated area of missing bone tissue extending from the interface between bone and implant [24]. Osteolyses were described as mixed when both linear and cystic osteolysis was present (Fig. 2).

Implants were identified as loosened when there was a continuous radiolucency surrounding the stem, with or without visible migration of the implant. The assessment of osteolytic changes and implant loosening were performed by one of the authors (NK), a consultant radiologist with more than 20 years of radiology experience.

#### Assessments of bone quality

**Fig. 3:**
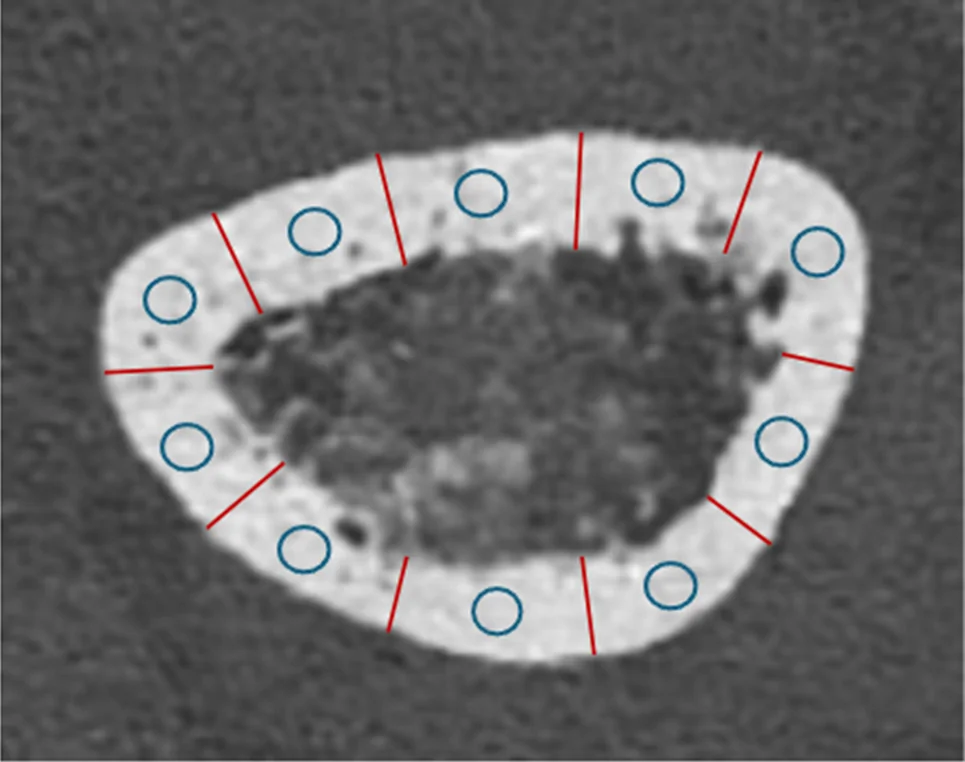
Axial image of distal radius with red lines representing measurements of cortical thickness, and blue circles denoting regions of interest (ROI) for calculation of average CT number (HU) in cortical bone

All measurements were performed by one of the authors (NK) in the polyenergetic (T3D) axial images, using a slice thickness of 1.0 mm, using manually placed regions of interest at ten different locations for each zone. Care was taken to place the measurements in similar positions. Zones R1, M1, and M2 were excluded because baseline cortical thickness was too limited for reliable measurements. Cortical thickness was measured as the shortest distance between the soft tissue-cortical bone interface and the cortical-trabecular bone interface. Cortical density was determined as the average CT number in circular regions of interest placed within the cortical bone (Fig. 3).

## Statistics

To analyze changes in osseointegration over time, we used ordinal logistic regression for correlated data based on generalized estimating equations (GEE). Examination was entered as a categorical predictor with the baseline examination (the day after surgery) as the reference level, allowing comparisons of 6 and 12 months versus baseline. To account for repeated measurements within the same patient, patient ID was used as the clustering variable. Observer was included as a fixed effect in the model.

To analyze changes in quantitative cortical quality measures, for each patient, at each examination and in each zone, the average cortical density and cortical thickness were calculated. Changes were assessed using a linear mixed-effects model with a heterogeneous first-order autoregressive covariance structure. Comparisons to baseline (0 months) were adjusted using a Sidak correction.

Interobserver agreement was assessed separately for each zone using quadratic-weighted Cohen’s kappa, which accounts for the ordinal nature of the scoring system. Kappa values were interpreted conventionally as follows: 0.41–0.60 moderate agreement, 0.61–0.80 substantial agreement, and > 0.80 almost perfect agreement. Two-sided *p*-values < 0.05 were considered statistically significant.

## Results

### Assessments of osseointegration and osteolysis

**Fig. 4:**
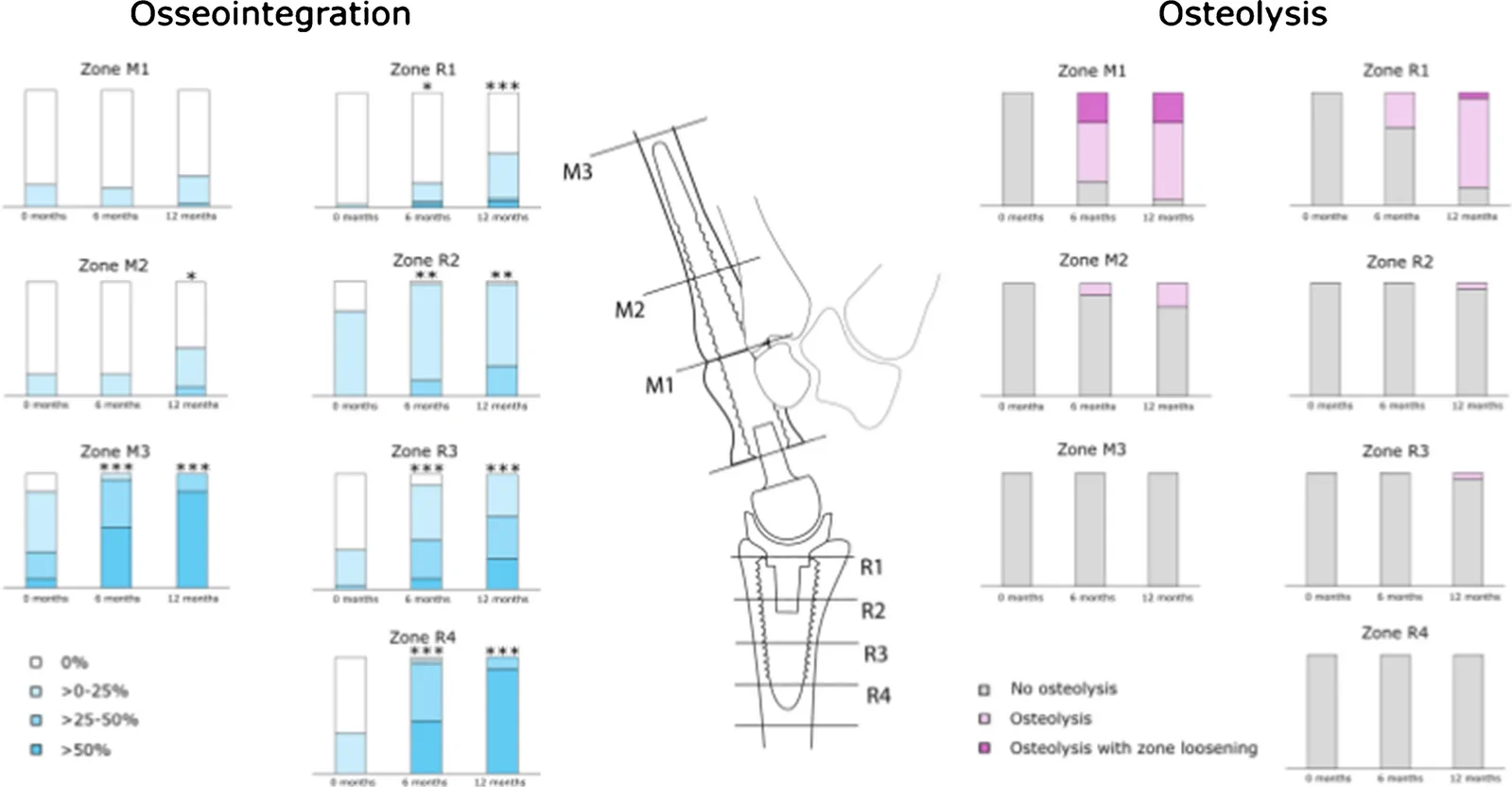
The diagrams on the left showing continuously increasing osseointegration of the implant in all zones but zone M1. The diagrams on the right show presence of osteolysis in the different zones, most frequent in the joint-near zones (M1 and R1). The bars show the proportion of patients with various degrees of cortical attachment/osseointegration or osteolysis at each time point. * Indicates significant differences between baseline and 6 and 12 months, respectively. * = *p* < 0.05; ** = *p* < 0.01; *** = *p* < 0.001

An overview of osseointegration and osteolysis in different zones at different time points is presented in Fig. 4.

## Osseointegration

Osseointegration of the implant increased in all zones except for in M1 during the 12-month follow-up period (Table 2). The highest percentage of osseointegration was found in the zones most distant from the joint space, with more than 50% of the circumference of the implant in 84.2% of the patients in zone M3 (around the metacarpal screw tip), and in 89.5% of the patients in zone R4 (around the radial screw tip) at 12 months.

**Table 2:** Number (proportion) of observations showing various degrees of osseointegration in different zones and at different time points

	Baseline				6 months				12 months				6 months vs. baseline		12 months vs. baseline	
*Zone*	*0%*	> *0–25%*	> *25–50%*	> *50%*	*0%*	> *0–25%*	> *25–50%*	> *50%*	*0%*	> *0–25%*	> *25–50%*	> *50%*	*OR (95% CI)*	*p*	*OR (95% CI)*	*p*
R1	37 (97.4%)	1 (2.6%)	0 (0%)	0 (0%)	30 (78.9%)	6 (15.8%)	1 (2.6%)	1 (2.6%)	20 (52.6%)	15 (39.5%)	1 (2.6%)	2 (5.3%)	10 (1.2–84)	0.035	34 (4.2–270)	0.001
R2	10 (26.3%)	28 (73.7%)	0 (0%)	0 (0%)	1 (2.6%)	32 (84.2%)	5 (13.2%)	0 (0%)	1 (2.6%)	27 (71.1%)	10 (26.3%)	0 (0%)	19 (2.4–153)	0.006	27 (3.4–213)	0.002
R3	25 (65.8%)	12 (31.6%)	1 (2.6%)	0 (0%)	3 (7.9%)	16 (42.1%)	11 (28.9%)	8 (21.1%)	0 (0%)	14 (36.8%)	14 (36.8%)	10 (26.3%)	27 (7.8–90)	** < **0.001	138 (17–1128)	** < **0.001
R4	25 (65.8%)	13 (34.2%)	0 (0%)	0 (0%)	1 (2.6%)	1 (2.6%)	19 (50%)	17 (44.7%)	0 (0%)	0 (0%)	4 (10.5%)	34 (89.5%)	741 (64–8533)	** < **0.001	NC	** < **0.001
M1	31 (81.6%)	7 (18.4%)	0 (0%)	0 (0%)	32 (84.2%)	6 (15.8%)	0 (0%)	0 (0%)	28 (73.7%)	9 (23.7%)	1 (2.6%)	0 (0%)	0.83 (0.25–2.8)	0.761	1.6 (0.55–4.8)	0.384
M2	31 (81.6%)	7 (18.4%)	0 (0%)	0 (0%)	31 (81.6%)	7 (18.4%)	0 (0%)	0 (0%)	22 (57.9%)	13 (34.2%)	3 (7.9%)	0 (0%)	1.0 (0.31–3.2)	> 0.999	3.4 (1.2–9.6)	0.021
M3	6 (15.8%)	20 (52.6%)	9 (23.7%)	3 (7.9%)	0 (0%)	2 (5.3%)	14 (36.8%)	22 (57.9%)	0 (0%)	0 (0%)	6 (15.8%)	32 (84.2%)	25.5 (8.0–81)	** < **0.001	90 (22–395)	** < **0.001

## Osteolytic changes

At 6 months, 31.6% (6 out of 19) of the patients exhibited osteolytic changes in zone R1 (the most distal zone of the radius); and at 12 months, 84.2% (16 patients). One of the patients, 5.3%, osteolysis was found in zones R2 and R3 at 12 months. In zone M1 (the capitate), there were 78.9% (15 patients) at 6 months, and 94.7% (18 patients) at 12 months. In zone M2 (the base of the metacarpal), 10.5% (2 patients) at 6 months, increasing to 21.1% (4 patients) at the 12-month follow-up. Patients with osteolytic changes at the 6-month examination all exhibited progressive osteolysis in at least one zone at 12 months.

In zone R1, 2 patients exhibited linear osteolysis, 10 patients cystic, and 4 patients a mix of linear and cystic osteolytic changes at 12 months. In one of the patients with cystic osteolysis in zone R1, the osteolytic changes continued into zones R2 and R3. In zone M1, 8 patients exhibited linear osteolysis, 4 patients cystic, and 6 patients a mix of linear and cystic osteolytic changes. In zone M2, 1 patient showed linear osteolysis, 2 patients cystic, and 1 patient a mix of linear and cystic osteolytic changes.

No loosening of the implant components was observed. In one patient, there was no remaining bone–implant contact in zone R1, and in five patients, radiolucent zones were present around the stem through the capitate, M1. However, the implants remained osseointegrated in more proximal radial zones and metacarpal zones, respectively.

## Interobserver reliability

Interobserver agreement was moderate to almost perfect across zones. Quadratic-weighted kappa values were 0.719 for R1, 0.595 for R2, 0.827 for R3, 0.898 for R4, 0.621 for M1, 0.830 for M2, and 0.809 for M3. Agreement was highest in R4 and lowest in R2.

## Assessments of bone quality

### Cortical thickness

A continuous significant reduction in cortical thickness was found in zones R2 and R3 after 6 and 12 months. No change was found in zones R4 and M3 (Table 3).

**Table 3:** Mean and SD of cortical thickness measured in different zones. Significant differences between baseline at 0 months, and 6 and 12 months respectively are indicated with *p*-value

	Baseline	6 months	12 months
Zone R2	1.4 ± 0.3	1.2 ± 0.3 *p* < 0.0001	1.1 ± 0.3 *p* < 0.0001
Zone R3	1.8 ± 0.4	1.7 ± 0.3 *p* = 0.012	1.6 ± 0.3 *p* < 0.0001
Zone R4	2.0 ± 0.3	2.0 ± 0.3 n.s	2.0 ± 0.4 n.s
Zone M3	1.4 ± 0.3	1.4 ± 0.2 n.s	1.4 ± 0.2 n.s

## Cortical density

In zone R2, a reduction in bone density measured as HU was found both after 6 and after 12 months. In zones R3, R4, and M3, there was a tendency for reduced bone density, even though the results were not significant (Table 4).

**Table 4:** Mean and SD of cortical attenuation value (HU) measured in different zones. Significant differences between baseline at 0 months, and 6 and 12 months respectively are indicated with *p*-value

	Baseline	6 months	12 months
Zone R2	1341 ± 124	1248 ± 98 *p* = 0.006	1251 ± 77 *p* = 0.009
Zone R3	1369 ± 115	1312 ± 58 n.s	1320 ± 42 n.s
Zone R4	1367 ± 107	1338 ± 38 n.s	1342 ± 34 n.s
Zone M3	1330 ± 112	1293 ± 46 n.s	1297 ± 43 n.s

## Discussion

In this study, we used PCD-CT to investigate bone changes appearing during the first year after total wrist arthroplasty. We evaluated osseointegration, periprosthetic osteolysis, and bone quality changes in terms of cortical thickness and density in different zones around the implants.

**Fig. 5:**
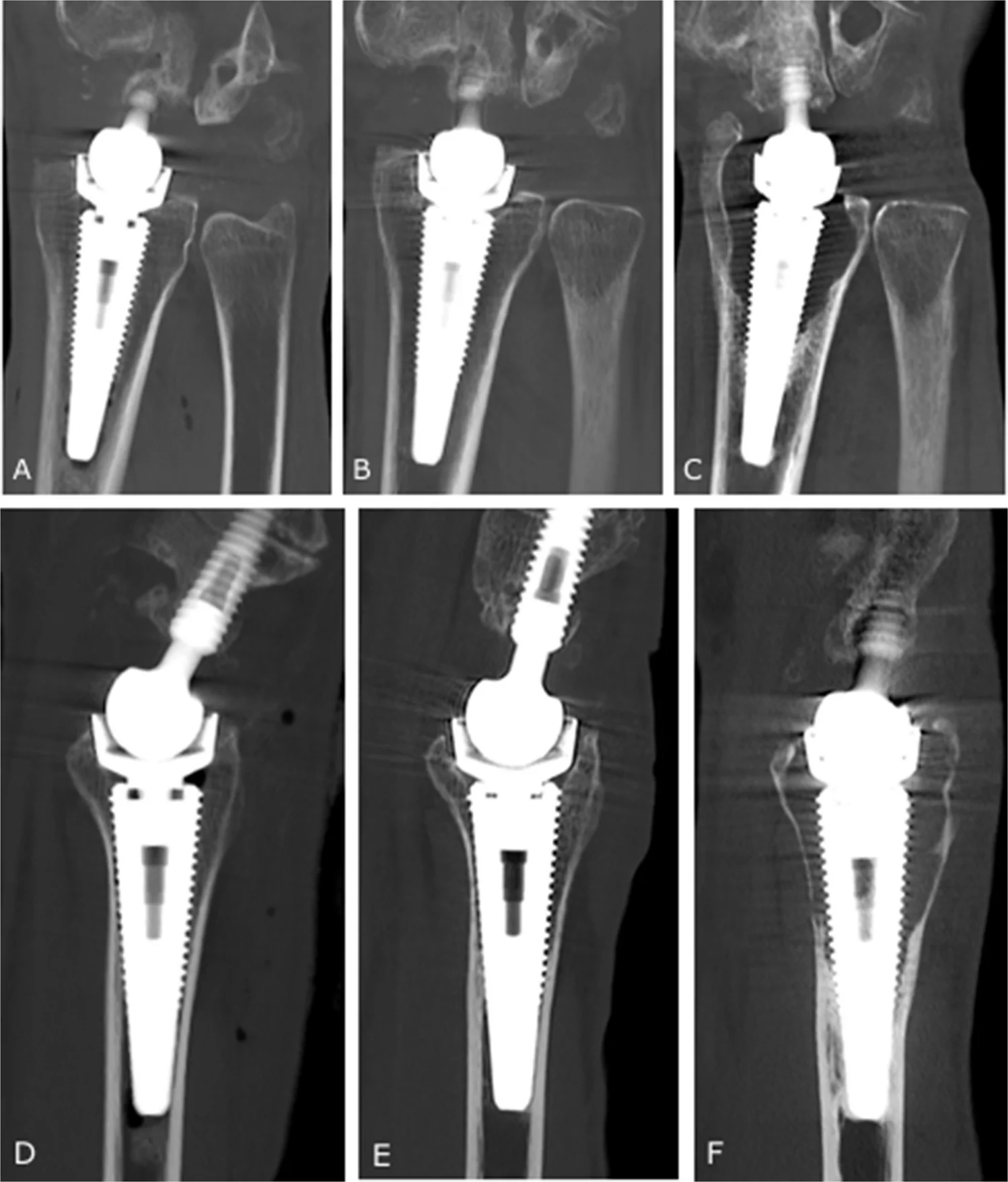
Development of cystic osteolysis in radius: **A** Coronal image of the wrist the day after surgery. **B** At 6 months, osteolysis has appeared at the most distal part of the screw. **C** At 12 months, cystic osteolysis has evolved. Concomitant development of osseointegration: **D** Sagittal image of the wrist the day after surgery. **E** At 6 months small amounts of trabecular bone are beginning to evolve from the endocortex towards the implant at the stem tip. **F** At 12 months, bone tissue surrounds the proximal part of the screw

The main finding was that PCD-CT revealed clear signs of continuous osseointegration to the implant surface, even though concomitant periprosthetic osteolysis was observed near the joint space in almost all patients at 12 months after surgery (Fig. 5).

We found that osteolysis started at the interface between bone and implant stem near the joint space (zones R1 and M1), progressing distally from the joint. Patients with osteolysis in a zone distal to the joint space concomitantly had osteolysis in the zone nearer the joint space. Osteolysis around the distal radial component was present in 16 out of 19 patients, and around the proximal metacarpal component in all but one of the patients at 12 months. This is a considerably higher proportion compared to the findings in a study by Holm-Glad et al. where 6 out of 20 patients with Motec TWA exhibited osteolysis around the radial component, and 9 patients around the metacarpal component at 12 months [2]. The higher number in our study might be due to different articulation materials. In their study, the Motec had a CoCr metal-on-metal ball and socket, whereas in our study there was an ultrahigh-molecular-weight-polyethylene (UHMPE) cup lining that possibly causes more inflammatory reactions to wear particles. Differences may also reflect the imaging modality used. In our study, PCD-CT provides high spatial resolution and three-dimensional assessment. This may have increased the sensitivity for detecting osteolysis in our cohort compared with the radiograph-based assessment used by Holm-Glad et al. [2].

In a study by Reigstad et al. 2017, loosening of implants demonstrated a rapid, linear osteolytic pattern, while juxta-articular scalloping (cystic) osteolysis halted after 1 to 2 years and did not correlate with component loosening [25]. We found both linear and cystic, as well as mixed osteolysis in patients exhibiting loss of bone attachment in the capitate (zone M1), and both linear and cystic, as well as mixed osteolytic changes progressing into the base of the corresponding metacarpal (M2). However, during the first 12 months postoperatively there was still osseointegration in the metacarpal, and no clinically relevant loosening as the primary fixation of the distal screw is in the isthmus area of the metacarpal bone (zone M3).

We evaluated bone quality changes in the different zones around the implant by comparing cortical density and cortical thickness at baseline, and at follow-ups.

The use of measuring mean CT number (HU) in a ROI in cortical bone is a simple method to estimate bone density as the attenuation of X-rays in CT is proportional to atomic mass and density in each voxel. Both bone density and cortical thickness reflect local stress changes [26, 27]. The implantation of a wrist prosthesis changes the distribution of mechanical forces in the joint [28]. Strain reduction induces bone resorption according to Wolff’s law.

We found reduced cortical thickness in the distal radial zones (R2 and R3), and a significant reduction in bone density in zone R2, suggesting reduced strain at the distal parts of the radial component. No changes were found at the radial (zone R4), or at the metacarpal stem tip (zone M3), which might be due to preserved (or increased) strain in these areas, maybe because this is where the implants get their primary fixation upon placement. These regions correspond to the areas where the screw has its largest cortical attachment.

Reduced bone density in areas around wrist implants has previously been assessed using DXA scans, and implant design has shown to have an impact on bone remodeling, resulting in bone resorption/stress shielding and changes in cortical thickness [2, 13, 29]. In an in vitro study by Completo et al. 2017, strain around a wrist implant was reduced at the distal radial component but increased at the radial stem tip [11]. It is plausible that the strain pattern is similar in bone around the radial component of the implants included in our study.

**Fig. 6:**
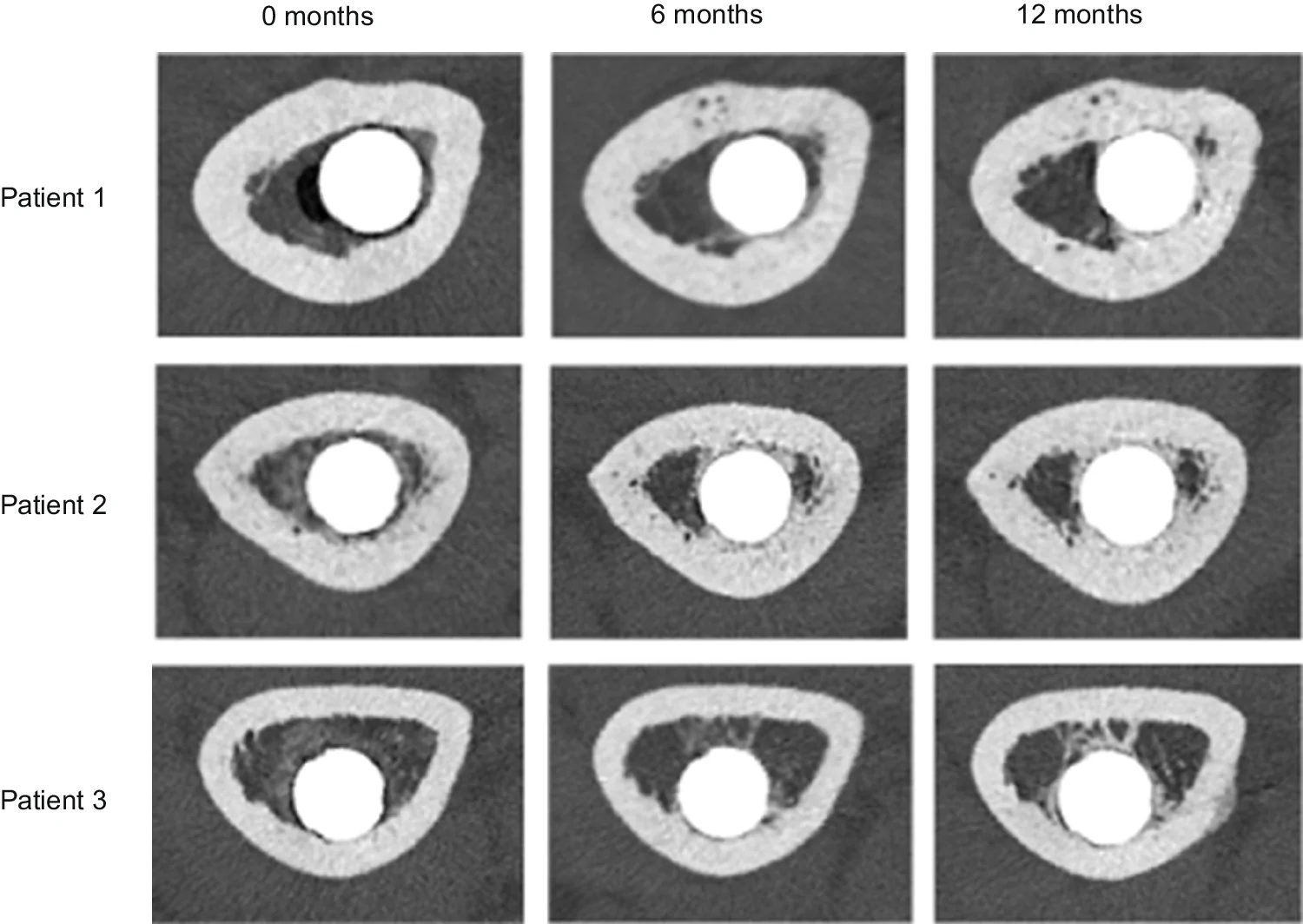
Axial images showing the distal radius near the stem tip (zone R4) in three patients. The day after surgery (0 months), there is diffuse high attenuation material in the medullary cavity consisting of blood and bone debris from surgery; at 6 months, incipient bone tissue formation from the endocortex towards the implant can be seen; at 12 months, bone tissue between the endocortex and the surface of the implant is clearly visible

Although there were considerable osteolytic changes and reduced bone quality in the periprosthetic bone distally around the radius component and proximally around the metacarpal component, there was no loosening of the implants during the follow-up period. Conversely, we found signs of increasing osseointegration in all zones except the capitate (M1) in terms of formation of additional trabecular bone, reaching out from the endocortical surface towards the surface of the implant (Fig. 6). These direct findings of osseointegration have previously been visualized and described by Thomson et al. in an animal model using micro-CT [30]. Due to the high spatial resolution of PCD-CT used in our study, we were able to visualize these findings in our patients with TWA.

Additionally, changes in osseointegration could be evaluated throughout the follow-up period. Osseointegration is usually assessed indirectly on conventional radiographic images and is defined by the absence of radiolucent zones (osteolysis) at the bone-implant interface, with or without changes in implant position at follow-up [31]. PCD-CT may provide complementary information by enabling more detailed evaluation of periprosthetic bone changes, thereby improving the evaluation of implant stability and advancing our understanding of the osseointegration process.

Our study has limitations. First, it is a single-center study with a limited number of patients included and only one type of wrist implant. Second, follow-up was limited to 12 months. Future studies should include larger cohorts, longer follow-up, and mechanical and functional outcomes to relate the imaging findings to long-term implant survival. Third, the assessment of bone quality was based on manual measurement of the cortex, since objective segmentation-based methods were found to be influenced by local density variations and trabecular bone. We therefore chose a standardized manual method with repeated measurements at 10 locations per zone and examination to improve precision. To conclude, PCD-CT can be used to detect bone changes important for implant stability after TWA. Foremost, we were able to visualize bone ingrowth to the implant over time. This happened while there was a concomitant reduction of bone quality in terms of reduced cortical density and thickness, as well as osteolytic changes around the implant parts closest to the joint space. By using PCD-CT we were able to observe this concomitant osseointegration important for assessment of implant stability.

## Data Availability

Upon reasonable request, data that support the findings of this study are available from Region Östergötland, Sweden. Restrictions may apply due to licensing and/or regulatory reasons. Contact the corresponding author for data requests.

## Abbreviations

CTDI_vol_: Volumetric computed tomography dose index
PCD-CT: Photon-counting CT
ROI: Region of interest
TWA: Total wrist arthroplasty
